# Gestational Trophoblastic Neoplasia Following Hydatidiform Mole and Non-Molar Pregnancy: Clinical and Prognostic Features from a 40-Year Cohort Study at a Reference Center in Southern Brazil

**DOI:** 10.3390/curroncol33060352

**Published:** 2026-06-11

**Authors:** Elza Maria Hartmann Uberti, Lidia Rosi de Freitas Medeiros, Rodrigo Bernardes Cardoso, Eduardo Silveira, Cassiano Burman Patias, Carlos Eduardo dos Santos Filho, Rosilene Jara Reis, Josenel Maria Barcelos Copetti, Jose Pio Furtado

**Affiliations:** 1 Gestational Trophoblastic Disease Center, Department of Obstetrics and Gynecology, Irmandade Santa Casa de Misericórdia de Porto Alegre, Porto Alegre 90035-074, RS, Brazil; elzahuberti@gmail.com (E.M.H.U.); rodrigobc@ufcspa.edu.br (R.B.C.); dredusilv@gmail.com (E.S.); cassiano.patias@santacasa.org.br (C.B.P.); carlosedu.medgo@gmail.com (C.E.d.S.F.); rosilene@ufcspa.edu.br (R.J.R.); josenel.copetti@gmail.com (J.M.B.C.); jpiofurtado@gmail.com (J.P.F.); 2 Department of Obstetrics and Gynecology, Federal University of Health Sciences of Porto Alegre, Porto Alegre 90050-170, RS, Brazil; 3 Guilherme Álvaro Hospital, Santos 11045-904, SP, Brazil; 4 Department of Radiology, Irmandade Santa Casa de Misericórdia de Porto Alegre, Porto Alegre 90020-090, RS, Brazil; 5 Department of Pathology, Federal University of Health Sciences of Porto Alegre, Porto Alegre 90050-170, RS, Brazil; 6 Department of Pathology, Irmandade Santa Casa de Misericórdia de Porto Alegre, Porto Alegre 90020-090, RS, Brazil; 7 Santa Rita Hospital, Irmandade Santa Casa de Misericórdia de Porto Alegre, Porto Alegre 90050-170, RS, Brazil

**Keywords:** gestational trophoblastic neoplasia, survival, choriocarcinoma, prognosis, reference center

## Abstract

Background: Gestational trophoblastic neoplasia (GTN) is rare but highly curable. This study compared clinical characteristics and outcomes between molar and non-molar disease. Methods: This retrospective cohort included 550 patients at a Brazilian reference center (1985–2025). Survival was assessed using Kaplan–Meier methods. Multivariable analyses included Poisson and Cox regression models. Results: Molar GTN comprised 86% of cases. Non-molar GTN (14%) presented with more advanced FIGO stages, higher WHO risk scores, and more metastases (*p* < 0.001). Overall five-year disease-specific survival (DSS) was 97.4% (95% CI 95.9–98.9) and progression-free survival (PFS) was 92.4% (95% CI 90.1–94.7). Non-molar disease had lower DSS (84.4% vs. 99.8%; *p* < 0.001) and PFS (85.3% vs. 93.5%; *p* = 0.049) in unadjusted analyses. However, after multivariable adjustment, GTN type was not independently associated with DSS (HR 9.41; 95% CI 0.70–127; *p* = 0.092) or PFS (HR 1.61; 95% CI 0.57–4.60; *p* = 0.372). Non-molar patients had a lower likelihood of subsequent pregnancy (RR 0.60; 95% CI 0.36–1.00; *p* = 0.049). Conclusions: Although non-molar GTN presents with more aggressive clinical features, survival outcomes appear to be primarily driven by baseline disease severity rather than GTN subtype itself.

## Simple Summary

Gestational trophoblastic neoplasia (GTN) is a rare but highly curable disease. In this study of 550 patients treated at a specialized reference center, non-molar GTN presented with more aggressive clinical features, including higher human chorionic gonadotropin levels, advanced FIGO stages, and more frequent metastases. Survival analysis showed excellent overall outcomes, although non-molar GTN had lower survival rates compared with molar disease. Multivariable analysis demonstrated that these differences were largely explained by baseline disease severity. These findings reinforce the importance of early diagnosis, risk stratification, and timely referral to specialized centers in the management of GTN.

## 1. Introduction

Gestational trophoblastic disease (GTD) comprises both premalignant and malignant trophoblastic disorders, whereas gestational trophoblastic neoplasia (GTN) refers specifically to the malignant entities, including invasive mole, choriocarcinoma, placental site trophoblastic tumor (PSTT), and epithelioid trophoblastic tumor (ETT) [1,2,3].

GTN encompasses malignant lesions arising from chorionic villi and extravillous trophoblasts [2]. Approximately 50% of GTN cases develop after a molar pregnancy, while the remaining follow non-molar gestations. In the latter, choriocarcinoma may arise after non-molar abortion, ectopic pregnancy, or term delivery, each accounting for roughly 25% of presentations [2,3]. PSTT and ETT are considerably less frequent and develop after term pregnancy or non-molar abortion in up to 95% of cases [1,2,3].

The incidence of choriocarcinoma in North America and Europe is estimated at 2 to 4 cases per 100,000 deliveries [4]. Higher incidence rates have been reported in Asian populations, ranging from 6 to 17 cases per 100,000 term pregnancies [5]. Advanced maternal age is a risk factor for choriocarcinoma [5]. Reliable epidemiologic data for the rare PSTT and ETT remain limited. However, according to the United Kingdom National Trophoblastic Disease Service, PSTT accounts for approximately 0.2% of all GTD cases [4]. Both PSTT and ETT may occur after term pregnancy, non-molar abortion, complete hydatidiform mole, or partial mole [1,2,5,6].

In both post-molar and non-molar GTN, histopathological confirmation is not mandatory. Diagnosis may be established based on clinical presentation and persistently elevated or rising serum human chorionic gonadotropin (hCG) levels, in accordance with established diagnostic criteria [1,2,3]. For the purposes of this study, non-molar GTN was defined as gestational trophoblastic neoplasia arising after a non-molar pregnancy, including term delivery, non-molar abortion, or ectopic pregnancy.

Once diagnostic criteria are met, patients should undergo comprehensive evaluation to determine anatomical staging, according to the International Federation of Gynecology and Obstetrics (FIGO, 2002) classification and risk-stratification scoring system [1,7,8,9]. These criteria are essential to predict resistance to single-agent chemotherapy and to guide initial multiagent treatment decisions [7,8]. This staging and scoring system provides a globally standardized framework for risk assessment and treatment selection [1,7,9] (Supplementary Table S1).

Since the introduction of effective chemotherapy in 1956, survival outcomes in GTN have improved substantially, largely due to early diagnosis and specialized management, with cure rates ranging from 80% to 90% [1,2,3]. Since the 1970s, international consensus has recommended that patients with rare forms of GTN—estimated at approximately 1 in 15,000 cases for invasive mole and 1 in 40,000 pregnancies for choriocarcinoma—should preferably be managed in specialized reference centers (RC) [2,10,11,12].

Despite sharing the same diagnostic framework and general treatment principles, molar and non-molar GTN may differ substantially in their clinical presentation, disease burden, treatment requirements, and prognosis [1,2,3]. Previous studies have suggested that patients with non-molar GTN are more likely to present with advanced disease, higher-risk scores, and metastatic involvement; however, the extent to which these differences independently affect survival outcomes remains uncertain [1,2,3]. Furthermore, data from large Latin American cohorts remain limited [5,6,7]. Therefore, comparing outcomes according to antecedent pregnancy may provide clinically relevant information for risk stratification, treatment planning, and patient counseling.

Therefore, this study aimed to compare the clinical characteristics, treatment patterns, survival outcomes, and reproductive outcomes of patients with molar and non-molar GTN treated at a specialized Brazilian reference center over a 40-year period. Additionally, we evaluated outcomes according to the site of initial management (reference center versus non-reference center).

## 2. Methods

### 2.1. Study Design and Setting

This study was conducted and reported in accordance with the Strengthening the Reporting of Observational Studies in Epidemiology (STROBE) statement [13]. This retrospective cohort study, conducted at the GTDC, Santa Casa de Porto Alegre (SCPA), Brazil, included consecutive patients diagnosed with gestational trophoblastic neoplasia (GTN) between March 1985 and March 2025.

**Figure 1:**
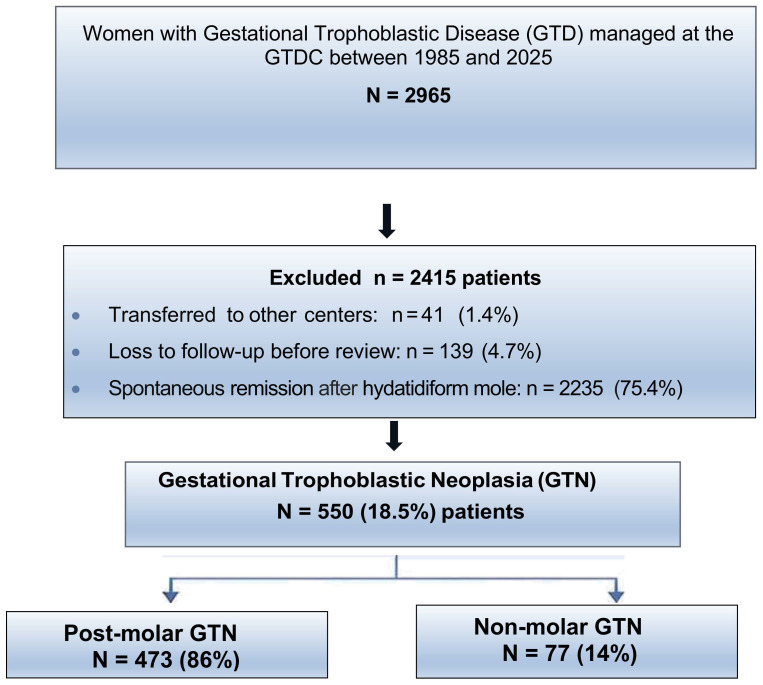
Flow diagram of patient selection from the Gestational Trophoblastic Disease (GTD) cohort and identification of patients with Gestational Trophoblastic Neoplasia (GTN) included in the study. **Abbreviations:** GTN = Gestational Trophoblastic Neoplasia [9]; TCD-RC/Santa casa de Porto Alegre-trophoblastic Disease center.

During the 40-year study period, 2965 women with Gestational Trophoblastic Disease (GTD) were managed and followed up at our GTDC by a multidisciplinary team under the same coordination. A total of 2416 patients were excluded from this study due to transfer to other RCs (42; 1.4%), loss to follow-up before GTD remission (139; 4.7%), or spontaneous remission of hydatidiform mole without progression to GTN (2235; 75.4%). Consequently, 550 patients (18.5%) with a confirmed GTN diagnosis were included in the final analysis (Figure 1). To ensure consistency across the 40-year study period, all patients were classified according to the FIGO 2002 staging and risk-scoring system, irrespective of the year of diagnosis. Patients diagnosed before 2002 were retrospectively staged and risk-classified using the clinical, laboratory, and imaging information available in their medical records.

This study evaluated the clinical characteristics, treatment patterns, and oncologic and reproductive outcomes of patients with GTN, with comparative analyses according to:

1. type of GTN (molar vs. non-molar); and
2. site of initial treatment (RC vs. outside RC).

### 2.2. Study Population

Patients were categorized as:

• Molar GTN (G1; *n* = 473)
• Non-molar GTN (G2; *n* = 77)

The primary exposure was GTN subtype (molar vs. non-molar), defined based on antecedent pregnancy and established clinical criteria.

A predefined subgroup analysis of choriocarcinoma cases (*n* = 71) compared outcomes by type of antecedent pregnancy (molar vs. non-molar), given the aggressive clinical behavior of this GTN subtype.

Additionally, cases were stratified by site of initial management: specialized care at our RC (*n* = 273) versus treatment at non-specialized services (non-RC; *n* = 277). For this analysis, initial management was defined as the healthcare service where GTN was first diagnosed and treatment was initiated before referral, when applicable.

**Inclusion criteria**

• Confirmed diagnosis of GTN
• Available clinical and treatment data
• Minimum follow-up of 12 months

**Exclusion criteria**

• Incomplete medical records preventing outcome evaluation
• Diagnosis revised to non-GTN condition

### 2.3. GTD Management and Patient Follow-Up

After uterine evacuation, patients underwent standardized follow-up at our GTDC, including serial serum hCG monitoring, clinical assessment, and transvaginal ultrasonography when clinically indicated. Histopathological specimens were reviewed whenever available. Serum hCG measurements were performed using the institutional laboratory assays available at the time of patient management [14,15].

### 2.4. Data Collection

Data were extracted from institutional medical records and cross-validated using oncology registry databases. hCG was measured using standardized laboratory assays, preferably at the GTDC.

Variables collected included:

• Demographic and obstetric characteristics
• Antecedent pregnancy
• Pretreatment hCG
• Histopathological diagnosis
• FIGO 2002 stage and WHO prognostic score
• Interval between antecedent pregnancy and treatment initiation
• Site of initial management (specialized reference center [RC] versus non-reference center [non-RC])
• Initial chemotherapy regimen and indication
• Response to first-line treatment
• Time to hCG normalization (<5 IU/L)
• Need for second-line therapy
• Surgical procedures
• Treatment-related complications
• Reproductive outcomes

**Outcomes**

Primary outcomes included:

• Disease-specific survival (DSS)
• Progression-free survival (PFS)

Survival time was calculated from the date of GTN diagnosis to the date of event (disease-related death or progression/recurrence) or to last follow-up.

Secondary outcomes included:

• Time to hCG normalization
• Treatment response rates
• Recurrence
• Need for second-line therapy
• Treatment-related complications
• Reproductive outcomes
• Impact of initial treatment site
• Impact of a choriocarcinoma diagnosis

### 2.5. Bias

This study has limitations inherent to its retrospective design. Selection and referral biases are expected, as patients treated at reference centers may not be representative of the general population, and those referred after initial management at a non-specialized service often present with more advanced disease. Information bias is a potential limitation arising from reliance on medical records, although data were systematically collected and cross-validated. Furthermore, the extensive study period may have introduced temporal biases reflecting changes in diagnostic and treatment protocols. Despite multivariable adjustment, residual confounding cannot be excluded. Finally, patients excluded due to transfer or loss to follow-up may have differed in disease severity, potentially introducing further selection biases.

### 2.6. Study Size

The sample included 550 patients with GTN, representing all eligible cases treated during the study period. Due to the use of convenience sampling, no formal sample size calculation was performed.

### 2.7. Statistical Analysis

Continuous variables were expressed as mean ± standard deviation (SD) or median and interquartile range (IQR), depending on distribution assessed using the Shapiro–Wilk test. Categorical variables were presented as absolute frequencies and percentages.

Groups were compared using:

• Student’s *t*-test or Mann–Whitney *U* test for continuous variables
• Chi-square test or Fisher’s exact test for categorical variables
• Adjusted residual analysis for contingency tables

Survival was estimated using the Kaplan–Meier method, and differences between curves were compared by the log-rank test. Multivariable Cox regression models controlled for confounders of primary outcomes. Secondary dichotomous outcomes were analyzed using multivariable Poisson regression with robust variance to estimate adjusted risk ratios. Multivariable linear regression evaluated time to hCG normalization after transformation, when necessary. Event-free patients were censored at last follow-up.

Potential confounders were predefined based on clinical relevance and literature, including age, pretreatment hCG, FIGO stage, WHO prognostic score, metastatic disease, initial treatment site, interval from antecedent pregnancy to treatment, and chemotherapy regimen. These variables were included in multivariable models to adjust for confounding.

Some variables, particularly FIGO stage and the prognostic risk score (FIGO 2002 system), were excluded from the multivariable models to avoid multicollinearity. All prespecified confounders were included simultaneously in the multivariable models unless collinearity was detected.

Statistical significance was defined as a two-sided *p* < 0.05; however, effect estimates and 95% confidence intervals (CI) were prioritized for clinical interpretation.

Collinearity between FIGO stage and the WHO prognostic score precluded their simultaneous inclusion in multivariable models. Separate models were constructed to prevent overadjustment and ensure the stability of estimates [9].

Complete-case analysis was performed, as missing data were negligible (<5%) and assumed to be missing at random. No variable had more than 5% missing data. Sensitivity analyses were performed using alternative multivariable models excluding collinear variables, with consistent results.

Statistical analyses were performed using IBM SPSS Statistics version 27.0 (IBM Corp., Armonk, NY, USA) [16].

### 2.8. Ethical Considerations

The study was approved by the Institutional Review Board of Santa Casa de Porto Alegre (SCPA) and the Federal University of Health Sciences of Porto Alegre (UFCSPA) and was registered in Plataforma Brasil (CAAE: 90723125.0.0000.5335). The study was conducted in accordance with institutional and national ethical standards for retrospective studies. Patient confidentiality was maintained, and all data were anonymized before analysis. The requirement for informed consent was waived due to the retrospective nature of the study.

## 3. Results

### 3.1. Patient Characteristics

A total of 550 patients (18.5%) with confirmed GTN were included in the final analysis (Figure 1), with none meeting the exclusion criteria. Mean age at diagnosis was 31.2 ± 9.3 years in both groups. Adolescents (≤19 years) accounted for 10.8% of patients; when stratified by groups, adolescents represented 10.8% in G1 and 3.9% in G2, with no statistically significant difference between groups (Table 1). Likewise, the proportion of patients aged ≥40 years did not differ significantly between groups.

Most patients (77.5%) were treated within the Brazilian public health system (SUS). Initial management at the GTDC was significantly more frequent in G1 than in G2 (54.8% vs. 18.2%, *p* < 0.001). Follow-up was completed for 92.7% of patients. Median follow-up duration was 49 months in G1 (IQR 23–66) and 50 months in G2 (IQR 25.5–121), with no significant difference between groups (*p* = 0.095) (Table 1).

### 3.2. Clinical Characteristics of GTN

The most common antecedent pregnancy was complete hydatidiform mole (71.3%), occurring predominantly in G1 (82.7%), whereas abortion and delivery were more frequent in G2 (54.5%; *p* < 0.001) (Table 1). Median pretreatment hCG was lower in G1 (7026 vs. 27,770; *p* < 0.001) (Table 1). Clinically, invasive moles predominated in G1 (93.2%), whereas choriocarcinoma was the most common diagnosis in G2 (62.3%) (*p* < 0.001) (Table 2). Other trophoblastic tumors were rare and, therefore, not evaluated. FIGO stage I was the most frequent in both groups, but the proportion was higher in G1 (87.3%) than in G2 (54.5%) (*p* < 0.001) (Table 2). Most G1 patients presented with FIGO stage I disease (95.6%) (Table 2).

According to the WHO prognostic score, 95.6% of G1 patients were classified as low risk, while 41.6% of G2 patients were high risk (*p* < 0.001) (Table 2) [9]. The median interval from antecedent pregnancy to treatment initiation was 7 weeks (IQR 5–11) in G1 and 14 weeks (IQR 6–25) in G2 (*p* < 0.001) (Table 1) [9].

### 3.3. Treatment Characteristics

As expected, single-agent chemotherapy predominated as initial treatment in G1. Methotrexate with folinic acid (MTX + FA) was used in 58.4% of G1 patients versus 23.4% in G2 (*p* < 0.01), while 31.5% of G1 patients received pulsed actinomycin-D (Table 3). Multiagent chemotherapy with EMA-CO (etoposide, methotrexate, dactinomycin, cyclophosphamide, vinblastine) was administered as first-line treatment in 2.5% of patients in G1 and 28.2% in G2 (*p* < 0.001), primarily in high-risk cases according to the prognostic scoring system (Table 3). No significant differences were found between groups regarding response to first-line chemotherapy (74.0% vs. 67.5%, *p* = 0.495). Median time to hCG normalization (<5 IU/L) was 8 weeks in G1 and 10 weeks in G2 (*p* = 0.200) (Table 3). Similarly, there were no significant differences in the median number of chemotherapy cycles required to achieve remission (4 in both groups; *p* = 0.437) or of consolidation cycles (2 in both groups; *p* = 0.294) (Table 3).

### 3.4. Metastatic Disease and Complications

Metastatic disease was significantly more frequent in G2 than in G1 (48.1% vs. 13.1%) (*p* < 0.001) (Table 2). The lung was the most common metastatic site (10.9%) (Table 2). Surgery was performed less frequently in G1 than in G2 (32.3% vs. 66.9%; *p* < 0.001) (Table 2). Hysterectomy was the most frequent intervention, with no significant difference between groups (36.6% vs. 52.0%; *p* > 0.05) (Table 2). Among the 203 surgically treated patients, 82 (40.4%) underwent hysterectomy. Thirty-three procedures (40.3%) were performed at the Reference Center, while 49 (59.7%) had been carried out before referral to the Center (Supplementary Table S4). At our Reference Center, the most common indications for hysterectomy were recurrent disease (15 cases, 45.5%), resistance to single-agent chemotherapy (5 cases, 15.2%), persistent hemorrhage associated with a uterine arteriovenous malformation (4 cases, 12.1%), placental site trophoblastic tumor (2 cases, 6.1%), patient preference (3 cases, 9.1%), and other indications (4 cases, 12.1%).

Conversely, repeat uterine evacuation was significantly more common in G1 than in G2 (39.2% vs. 16.0%; *p* = 0.003) (Table 2). Additional procedures, such as laparotomy for other indications, were significantly more common in G2 (20.0% vs. 4.6%; *p* = 0.003) (Table 2). Clinical complications occurred in 49.3% of the 294 patients treated with methotrexate and in 69.6% of the 171 patients treated with actinomycin D; all were mild (Supplementary Table S2). The most frequent complications were stomatitis (71.7% vs. 18.5%) and nausea, vomiting, and diarrhea (22.1% vs. 27.7%) in the methotrexate and actinomycin D groups, respectively (Supplementary Table S2). Specific methotrexate-related toxicities included dry eye (26.2%), pleuritic pain (22%), and hepatotoxicity (10.3%). Among the 45 patients receiving multiagent chemotherapy, the most common complications were alopecia (100%), neutropenia (91%), thrombocytopenia (22.2%), and severe anemia (28.9%).

### 3.5. Second-Line Treatment and Recurrence

Second-line treatment, primarily indicated for chemoresistance, was administered at similar frequencies in G1 and G2 (69% vs. 60%; *p* = 0.818) (Table 4). Pulsed actinomycin-D was the predominant second-line regimen, used significantly more frequently in G1 than in G2 (52.8% vs. 15.4%; *p* < 0.001), followed by methotrexate (24.5%) (Table 4). No significant differences in response to second-line chemotherapy were observed between G1 and G2 (73.2% vs. 63.0%; *p* = 0.260) (Table 4).

Post-treatment recurrence was low and comparable between G1 and G2 (5.7% vs. 11.7%; *p* = 0.116), similar to previously reported data [1,2,4]. At recurrence, median hCG levels were similar between groups, at 27 IU/L (IQR 11–206) (Table 4).

Recurrence management frequently involved hysterectomy with or without chemotherapy, at similar rates between G1 and G2 (44.4% vs. 33.3%; *p* = 0.204). Overall, 15 deaths (2.7%) were recorded, 7 of which (1.3%) were attributable to GTN. These proportions were significantly higher in G2 than in G1 (4.6% vs. 0.2%; *p* < 0.001). This corresponds to an absolute risk difference of 5.8%, highlighting the clinically relevant increase in mortality among patients with non-molar GTN. Among these patients, the mean age at death was 38.8 ± 9.4 years (Table 4).

### 3.6. Reproductive Outcomes

Favorable reproductive outcomes were observed following remission in both groups among the 197 patients with known pregnancy outcomes, with significant difference between groups (G1: 39.3% vs. G2: 14.3%; *p* < 0.001) (Supplementary Table S3). Among women who did not conceive again, the most common reasons were prior hysterectomy (G1: 37.3% vs. G2: 64.8%), advanced maternal age or completed parity (G1: 24.0% vs. G2: 11.0%), and personal decision (G1: 21.6% vs. G2: 3.7%); all of these differences were statistically significant (*p* < 0.001) (Supplementary Table S3).

### 3.7. Disease-Specific Survival and Progression-Free Survival According to GTN Type

In this cohort, GTN-related mortality was significantly higher among patients with non-molar GTN.

**Figure 2:**
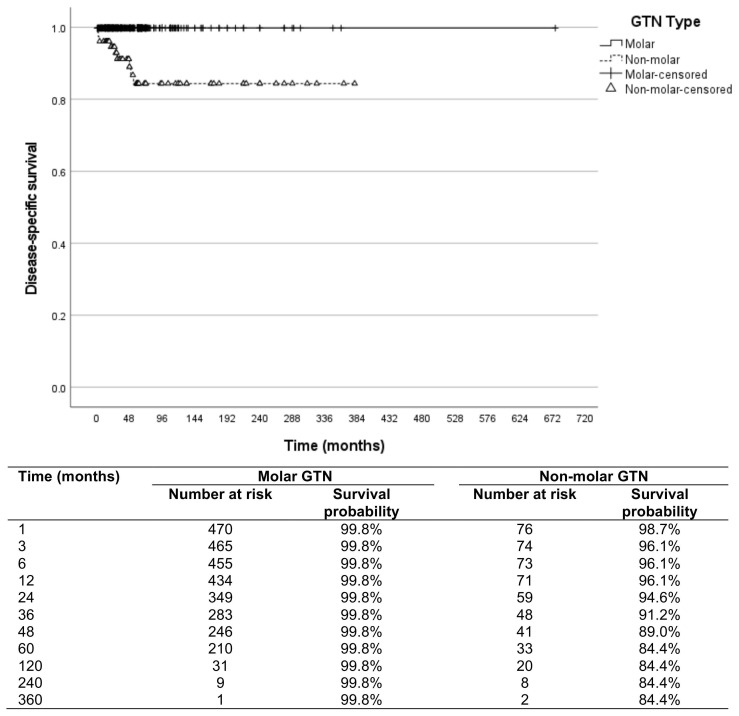
Disease-specific survival according to GTN type (log rank test: c^2^ = 46.8; *p* < 0.001). **Abbreviation:** GTN = Gestational Trophoblastic Neoplasia.

**Figure 3:**
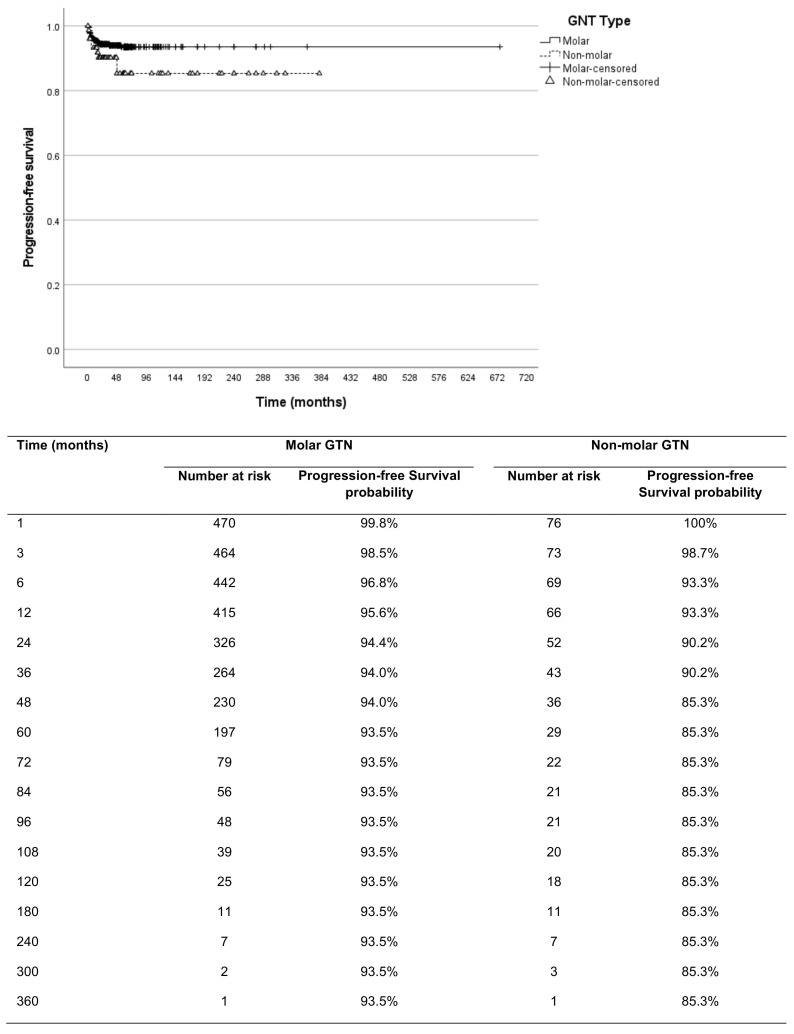
Progression-free survival according to GTN type (log rank test: c^2^ = 3.89; *p* = 0.049). **Abbreviation:** GTN = Gestational Trophoblastic Neoplasia.

Kaplan–Meier analysis demonstrated significantly worse disease-specific survival (DSS) in patients with non-molar GTN than in those with molar GTN (log-rank χ^2^ = 46.8; *p* < 0.001) (Figure 2). While DSS remained stable at 99.8% over time in molar GTN, it declined progressively in the non-molar group, reaching approximately 85.3% in 5 years. Similarly, progression-free survival (PFS) was significantly lower in the non-molar group (log-rank χ^2^ = 3.89; *p* = 0.049) (Figure 3).

In unadjusted analyses, non-molar GTN was associated with need for surgery (RR 2.01; 95% CI 1.63–2.48) and a markedly increased risk of disease-specific mortality (HR 54.0; 95% CI 6.85–426). However, the wide confidence interval suggests substantial imprecision, likely attributable to the low number of events and imbalances between groups (Table 5).

After adjustment for relevant confounders—including pretreatment hCG levels, WHO risk score, metastatic status, treatment modality, and interval from antecedent pregnancy—GTN subtype was no longer independently associated with DSS (adjusted HR 9.41; 95% CI 0.70–127; *p* = 0.092) and PFS (HR 1.61; 95% CI 0.57–4.60; *p* = 0.372) (Table 5).

Notably, reproductive outcome was the only parameter that remained independently associated with GTN subtype. Patients with non-molar GTN had a significantly lower likelihood of subsequent pregnancy (adjusted RR 0.60; 95% CI 0.36–1.00; *p* = 0.049), highlighting the long-term reproductive impact of more aggressive disease and treatment (Table 5).

### 3.8. Impact of Initial Treatment Site

Among the cohort, 273 patients (49.6%) received initial treatment at the GTDC, and 277 (50.4%) at external services (Supplementary Table S4). Patients initially treated elsewhere presented with more advanced disease, including higher pretreatment hCG levels, FIGO stage, and WHO risk score (Supplementary Table S4). Patients initially treated elsewhere presented with higher pretreatment hCG levels, more advanced FIGO stages, higher WHO risk scores, and a longer interval between antecedent pregnancy and treatment initiation. These patients also had a longer interval between the antecedent pregnancy and treatment initiation. Patients treated at the GTDC were more likely to receive single-agent chemotherapy, whereas those referred from external services required multiagent regimens and surgical interventions more frequently (Supplementary Table S4). Metastatic disease was also more common among patients initially treated outside the GTDC (Supplementary Table S4). Despite these differences in disease severity and treatment patterns, disease-specific survival did not differ significantly between groups (Supplementary Figure S1). Progression-free survival was slightly lower among patients initially treated at the GTDC, but the difference between groups was not statistically significant (Supplementary Figure S2).

### 3.9. Choriocarcinoma Subgroup

A subgroup analysis was performed for the 71 patients diagnosed with choriocarcinoma, including 23 patients in G1 (32.4%) and 48 in G2 (67.6%; *p* < 0.001) (Supplementary Table S5). Patients in the molar group were more frequently managed at the GTDC from the onset, whereas most non-molar cases received initial treatment at non-specialized centers (*p* = 0.003). Non-molar GTN was associated with significantly higher pretreatment hCG levels (median 53,700 vs. 19,106 IU/L; *p* = 0.002) and a greater prevalence of WHO high-risk disease (58.3% vs. 21.7%; *p* < 0.001) (Supplementary Table S5). The frequency of surgical intervention did not differ significantly between molar and non-molar choriocarcinoma (65.2% vs. 75.0%, respectively; *p* = 0.565). Regarding chemotherapy, single-agent methotrexate was more frequent in molar GTN, whereas multiagent regimens, particularly EMA-CO, were more frequently used in non-molar cases (*p* = 0.021) (Supplementary Table S5). Despite these differences, survival outcomes remained excellent for all patients managed at our GTDC (Supplementary Table S5).

### 3.10. Analysis of Survival Outcomes (DSS and PFS) in the Overall Cohort

Kaplan–Meier survival analysis demonstrated excellent long-term outcomes in this cohort. Disease-specific survival was 99.3% at 12 months, remaining high at 97.4% after 60 months (Supplementary Figure S3). Survival curves plateaued after five years, reflecting sustained remission across the study population.

Similarly, progression-free survival was 95.3% at 12 months and 92.4% at 60 months. Most progression events occurred within the first two years of follow-up, after which the survival curve stabilized. These findings suggest that a minimum two-year follow-up is appropriate for patients with low-risk GTN (Supplementary Figure S4).

## 4. Discussion

In this large retrospective cohort of 550 patients with gestational trophoblastic neoplasia (GTN), we observed excellent long-term outcomes, with disease-specific survival exceeding 97% at five years. These findings confirm that GTN remains a highly curable malignancy when managed using standardized protocols and risk-adapted strategies [1,2,3,7,11]. However, prognosis varies according to clinical presentation and disease subtype. These findings indicate that the poorer outcomes observed in non-molar GTN are primarily driven by baseline disease severity rather than intrinsic biological differences.

After more than four decades of clinical experience at the Gestational Trophoblastic Disease Center (GTDC), a specialized reference center at Santa Casa de Porto Alegre (SCPA), providing multidisciplinary care to a large patient cohort maintained under stable medical leadership, our team evaluated the clinical outcomes of post-molar and non-molar GTN, comparing their clinical presentation and prognostic characteristics. Additionally, one analyzed treatment outcomes according to the site of initial management, specifically comparing patients who received primary care at a specialized RC with those initially treated outside such centers. To our knowledge, this study represents one of the largest single-center cohorts comparing molar and non-molar GTN in Latin America and one of the few studies to evaluate survival, reproductive outcomes, and the impact of initial treatment site within a 40-year follow-up period. Most cases in our cohort were post-molar GTN, consistent with the established epidemiology of the disease [1,2,3,5]. Routine monitoring of serum hCG levels after molar pregnancy plays a critical role in the early diagnosis of GTN and timely treatment initiation, contributing to high cure rates [1,2,3]. Current guidelines emphasize the importance of FIGO staging and WHO risk scoring in guiding treatment decisions [1,9].

The majority of patients presented with low-risk disease and achieved remission with single-agent chemotherapy. Methotrexate with folinic acid and pulsed actinomycin-D were the most commonly used regimens, with outcomes comparable to those reported in other reference centers [7,17,18]. These findings reinforce the effectiveness of single-agent therapy in appropriately selected patients. Pulsed actinomycin-D also remains a valuable second-line option following methotrexate resistance [17,18,19].

Non-molar GTN was associated with a more advanced clinical presentation, including higher pretreatment hCG levels, higher FIGO stages, and increased metastatic disease. This likely reflects delayed diagnosis, as routine hCG surveillance is not performed after non-molar pregnancies [1,2,3,4]. As a result, these patients more frequently required multiagent chemotherapy and surgical interventions [1,2,19]. The low recurrence rates observed in our cohort are consistent with previous reports. [1,2,4].

Despite these differences, overall survival outcomes remained favorable [1,2,3]. In unadjusted analyses, non-molar GTN was associated with worse survival. However, after adjustment for key confounders—including pretreatment hCG, WHO risk score, metastasis, treatment modality, and interval from antecedent pregnancy—GTN subtype was no longer independently associated with survival. These findings suggest that prognosis is primarily driven by baseline disease severity rather than intrinsic tumor biology.

Survival estimates should be viewed cautiously due to the small number of events, particularly in the non-molar group. This is reflected in the wide confidence intervals in multivariable analyses, indicating reduced precision. The large hazard ratios observed in unadjusted models likely reflect sparse events and baseline imbalances and should, therefore, be interpreted with caution.

Reproductive outcomes were generally favorable. A substantial proportion of patients achieved subsequent pregnancies after remission, consistent with prior reports [7,20]. However, patients with non-molar GTN were less likely to conceive, likely due to more intensive treatment and higher rates of hysterectomy [20].

The subgroup analysis of choriocarcinoma further supports these findings, demonstrating a more aggressive profile in non-molar cases. These patients presented with higher hCG levels and a greater proportion of high-risk disease. Previous studies have similarly shown that choriocarcinoma often requires multiagent chemotherapy, particularly in high-risk settings [1,2,3,11,19]. Nevertheless, survival remained favorable when patients were managed in a specialized center [21].

The site of initial treatment also influenced disease presentation. Patients treated outside the reference center presented with more advanced disease, including higher hCG levels and increased metastatic burden. These findings should be interpreted cautiously, as referral bias and differences in baseline disease severity likely contributed to the observed results. Although our data cannot establish a causal effect of treatment site, they are consistent with previous literature supporting early referral and management in specialized centers [8,21,22,23,24]. Because of the retrospective observational design, comparisons according to the site of initial management should be interpreted as descriptive associations rather than evidence of a causal effect of treatment location on outcomes.

The delayed presentation observed among patients with non-molar GTN is likely multifactorial. Unlike post-molar GTN, for which routine hCG surveillance is universally recommended after molar evacuation, women following term pregnancy, spontaneous abortion, or ectopic pregnancy are not routinely monitored with serial hCG measurements. Consequently, the diagnosis of non-molar GTN often depends on the recognition of non-specific symptoms such as abnormal uterine bleeding, persistent postpartum bleeding, respiratory complaints, or neurological manifestations secondary to metastatic disease. This diagnostic pathway may contribute to longer intervals between antecedent pregnancy and treatment initiation, higher pretreatment hCG levels, and more advanced FIGO stages at presentation, as observed in our cohort and in previous reports [1,2,3,4,8]. These findings may have particular relevance in low- and middle-income countries, where access to specialized trophoblastic disease centers and timely diagnostic evaluation may be limited. Although universal hCG surveillance after all pregnancies is unlikely to be feasible, increasing awareness among obstetricians, gynecologists, and primary care physicians regarding the possibility of GTN after non-molar gestations may facilitate earlier diagnosis and referral. Strategies aimed at improving referral pathways and access to specialized care could potentially reduce disease burden at presentation and further improve outcomes in these settings [8,21,22,23,24].

This study has several strengths. The large, well-characterized cohort with more than four decades of follow-up provides robust real-world data on a rare disease. Care was delivered within a specialized reference center under consistent clinical leadership, ensuring longitudinal uniformity in diagnostic and therapeutic protocols. This comprehensive dataset enabled a detailed evaluation of treatment patterns, survival outcomes, and reproductive outcomes, thereby enhancing the clinical relevance and generalizability of our findings.

Despite these strengths, some limitations warrant consideration. Another limitation relates to the 40-year study period, during which diagnostic criteria, imaging modalities, supportive care practices, and treatment strategies evolved substantially. Although FIGO recommendations changed over time, all patients included in this cohort were retrospectively staged and risk-classified according to the FIGO 2002 system, thereby enhancing comparability across the study period. In addition, patient management was conducted within the same specialized reference center under stable clinical leadership and largely consistent treatment principles. Nevertheless, residual temporal bias cannot be completely excluded, and changes in diagnostic and therapeutic practices over time may have influenced some observed differences between groups. Information regarding diagnostic pathways, time from GTN diagnosis to treatment initiation, and adherence to treatment guidelines at non-reference centers was not consistently available and therefore could not be systematically evaluated. Finally, although multivariable adjustment was performed, residual confounding cannot be excluded, particularly given the small number of events in the non-molar group.

Although the generalizability of these findings may be limited, the comparison between patients treated within and outside a reference center highlights important disparities in care and reinforces the need for early diagnosis and appropriate risk stratification. These findings support the continued evaluation of specialized referral systems for GTN management.

Future studies should focus on prospective multicenter cohorts to further evaluate prognostic factors and treatment outcomes in patients with non-molar GTN, particularly those with choriocarcinoma and other rare histological subtypes. In addition, studies exploring strategies to promote earlier diagnosis, optimize referral pathways to specialized centers, and improve long-term reproductive outcomes are warranted. Given the rarity of these conditions, international collaborative research efforts may provide more robust evidence to refine risk stratification and treatment approaches.

## 5. Conclusions

GTN is a highly curable malignancy when managed according to standardized protocols. Non-molar GTN and choriocarcinoma are associated with more advanced disease at presentation and a greater need for multiagent chemotherapy. Although the present study cannot establish a causal effect of centralized care systems, our findings are consistent with previous evidence supporting early diagnosis, appropriate risk stratification, and timely referral to specialized trophoblastic disease centers.

## Figures and Tables

**Table 1:** Sociodemographic and disease characteristics according to GTN type.

Variable	Total (*n* = 550)	Molar GTN (*n* = 473)	Non-Molar GTN (*n* = 77)	*p*-Value
	*n* (%)	*n* (%)	*n* (%)	
**Age (years, mean ± SD)**	31.2 ± 9.3	31.2 ± 9.4	31.2 ± 8.7	0.985 ^a^
**Age group**				0.072 ^b^
≤19 years	54 (9.8)	51 (10.8)	3 (3.9)	
20–39 years	395 (71.8)	332 (70.2)	63 (81.8)	
≥40 years	101 (18.4)	90 (19.0)	11 (14.3)	
**Place of initial treatment**				**<0.001** ^b^
Reference GTDC	273 (49.6)	259 (54.8)	14 (18.2)	
Outside GTDC	277 (50.4)	214 (45.2)	63 (81.8)	
**Pretreatment hCG (IU/L), median (IQR)**	8442 (1740–32,163)	7026 (1605–26,740)	27770 (3358–1,300,022)	**<0.001** ^c^
**Time from last pregnancy to initial treatment (weeks), median (IQR)**	8 (5–12)	7 (5–11)	14 (6–25)	**<0.001** ^c^
**Follow-up time (months), median (IQR)**	49.5 (23–66)	49 (23–66)	50 (25.5–121.5)	0.095 ^c^
**Pregnancy of origin**				**<0.001** ^b^
Complete mole	392 (71.3)	391 (82.7) *	1 (1.3)	
Partial mole	75 (13.6)	73 (15.4) *	2 (2.6)	
Abortion	47 (8.5)	5 (1.1)	42 (54.5) *	
Delivery	27 (4.9)	0 (0.0)	27 (35.1) *	
Ectopic	5 (0.9)	1 (0.2)	4 (5.2) *	
Unknown	4 (0.7)	3 (0.6)	1 (1.3)	
**Clinical outcomes at discharge**				**<0.001** ^d^
Discharged (persistent remission)	510 (92.7)	444 (93.9) *	66 (85.7)	
Post-remission loss to follow-up	24 (4.4)	22 (4.6)	2 (2.6)	
**Health insurance coverage**				0.967 ^c^
Public health system	426 (77.5)	367 (77.6)	59 (76.6)	
Private/other insurance	124 (22.5)	106 (22.4)	18 (23.4)	

^a^ Student’s *t*-test; ^b^ chi-square test; ^c^ Mann–Whitney test; ^d^ Fisher’s exact test. * Statistically significant based on adjusted residuals (*p* < 0.05). Bold *p*-values indicate statistical significance. **Abbreviations:** GTN = gestational trophoblastic neoplasia; GTDC = gestational trophoblastic disease center; IQR = Interquartile Range; hCG = human chorionic gonadotropin; SD = standard deviation.

**Table 2:** GTN characteristics and oncologic outcomes according to GTN type.

Variable	Total (*n* = 550)	Molar GTN (*n* = 473)	Non-Molar GTN (*n* = 77)	*p*-Value ^a^
	*n* (%)	*n* (%)	*n* (%)	
**FIGO stage (2002)**				**<0.001**
I	455 (82.7)	413 (87.3) *	42 (54.5)	
II	12 (2.2)	6 (1.3)	6 (7.8) *	
III	66 (12.0)	48 (10.1)	18 (23.4) *	
IV	17 (3.1)	6 (1.3)	11 (14.3) *	
**WHO risk score (2002)**				**<0.001**
Low (0–4)	453 (82.4)	423 (89.4) *	30 (39.0)	
Low (5–6)	38 (6.9)	29 (6.1)	9 (11.7)	
High (7–12)	52 (9.5)	20 (4.2)	32 (41.6) *	
Ultra-high	7 (1.3)	1 (0.2)	6 (7.8) *	
**Metastasis**				**<0.001**
No	451 (82.0)	411 (86.9) *	40 (51.9)	
Yes	99 (18.0)	62 (13.1)	37 (48.1)	
Lung	60 (10.9)	45 (9.5)	15 (19.5) *	
Vagina	5 (0.9)	3 (0.6)	2 (2.6)	
Multiple	31 (5.6)	12 (2.5)	19 (24.7) *	
Other site	3 (0.5)	2 (0.4)	1 (1.3)	
**Surgery**	203 (36.9)	153 (32.3)	50 (66.9)	**<0.001**
**Type of surgery**				**0.003**
Hysterectomy	82 (40.4)	56 (36.6)	26 (52.0)	
Repeat uterine evacuation	68 (33.5)	60 (39.2) *	8 (16.0)	
Hysteroscopy	8 (3.9)	7 (4.6)	1 (2.0)	
Laparotomy (other indication)	17 (8.4)	7 (4.6)	10 (20.0) *	
Embolization	4 (2.0)	4 (2.6)	0 (0.0)	
Pulmonary resection	1 (0.5)	1 (0.7)	0 (0.0)	
Neurosurgery	2 (1.0)	2 (1.3)	0 (0.0)	
**Final histopathological diagnosis**				**<0.001**
Invasive mole	458 (83.3)	441 (93.2) *	17 (22.1)	
CCA	71 (12.9)	23 (4.9)	48 (62.3) *	
PSTT	6 (1.1)	3 (0.6)	3 (3.9) *	
ETT	3 (0.5)	0 (0.0)	3 (3.9) *	
Same as initial histology	2 (0.4)	0 (0.9)	2 (2.6) *	
Other	10 (1.8)	6 (1.3)	4 (5.2) *	

^a^ Chi-square test * Statistically significant based on adjusted residuals (*p* < 0.05). Bold *p*-values indicate statistical significance. **Abbreviation:** GTN = gestational trophoblastic neoplasia; CCA = choriocarcinoma; ETT = epithelioid trophoblastic tumor; PSTT = placental site trophoblastic tumor; FIGO = International Federation of Gynecology and Obstetrics; WHO = World Health Organization.

**Table 3:** Treatment characteristics according to GTN type.

Variable	Total (*n* = 550)	Molar GTN (*n* = 473)	Non-Molar GTN (*n* = 77)	*p*-Value
**Initial CTx regimen**				**<0.001** ^a^
MTX/FA	294 (53.5)	276 (58.4) *	18 (23.4)	
Actinomycin D (pulse)	169 (30.7)	149 (31.5)	20 (26.0)	
Actinomycin D (5-day)	2 (0.4)	2 (0.4)	0 (0.0)	
EMA-CO	34 (6.2)	12 (2.5)	22 (28.6) *	
MAC III (MTX + Act-D + cyclophosphamide)	5 (0.9)	5 (1.1)	0 (0.0)	
Other MTX regimen	9 (1.6)	8 (1.7)	1 (1.3)	
Low-dose EP	6 (1.1)	1 (0.2)	5 (6.5) *	
EMA-EP	3 (0.5)	0 (0.0)	3 (3.9) *	
EMA	3 (0.5)	2 (0.4)	1 (1.3)	
No chemotherapy	23 (4.2)	17 (3.6)	6 (7.8)	
Other	2 (0.4)	1 (0.2)	1 (1.3)	
**Indication for chemotherapy**				**<0.001** ^a^
No CTx	11 (2.0)	8 (1.7)	3 (3.9)	
Standard MTX/FA	281 (51.1)	265 (56.0) *	16 (20.8)	
Act-D bolus	113 (20.5)	102 (21.6)	11 (14.3)	
Perioperative	20 (3.6)	18 (3.8)	2 (2.6)	
High-risk (multiagent chemotherapy)	48 (8.7)	21 (4.4)	27 (35.1) *	
Emergency (bleeding)	44 (8.0)	37 (7.8)	7 (9.1)	
Ultra-high risk	8 (1.5)	1 (0.2)	7 (9.1) *	
Other	25 (4.5)	21 (4.4)	4 (5.2)	
**Response to first-line treatment**				0.495 ^a^
No	136 (24.7)	113 (23.9)	23 (29.9)	
Yes	402 (73.1)	350 (74.0)	52 (67.5)	
No chemotherapy	12 (2.2)	10 (2.1)	2 (2.6)	
**Time to hCG normalization (weeks), median (IQR)**	9 (6–14)	8 (5–14)	10 (6–14)	0.200 ^b^
**Number of CTx cycles to remission, median (IQR)**	4 (2–6)	4 (2–6)	4 (3–6)	0.437 ^b^
**Number of consolidation cycles, median (IQR)**	2 (1–3)	2 (1–3)	2 (2–3)	0.294 ^b^

Footnotes: Data are presented as number (percentage) or median (interquartile range).^a^ Chi-square test; ^b^ Mann–Whitney test. * Statistically significant based on adjusted residuals (*p* < 0.05). Bold *p*-values indicate statistical significance. **Abbreviations**: Act-D = actinomycin D; CTx = Chemotherapy; EP = etoposide and cisplatin; EMA = etoposide/methotrexate; EMA-CO = etoposide, methotrexate, dactinomycin, cyclophosphamide, vinblastine; EMA-EP = etoposide, methotrexate, dactinomycin/etoposide, cisplatin; GTN = gestational trophoblastic neoplasia; normal; IQR = interquartile range; normal hCG = Human chorionic gonadotropin < 5 UI/L; MAC III = MTX + Act-D + cyclophosphamide; MTX/FA= methotrexate/folinic acid.

**Table 4:** Second-line Therapy and Outcomes According to GTN Type.

Variable	Total (*n* = 550)	Molar GTN (*n* = 473)	Non-Molar GTN (*n* = 77)	*p*-Value
**Pretreatment hCG (second-line)**	366 (31–4035)	307 (30–3881)	870 (95–5210)	0.388 ^a^
**Second-line CTx regimen**				**<0.001** ^b^
Actinomycin D (pulse)	70 (46.4)	66 (52.8) *	4 (15.4)	
Actinomycin D (5-day)	7 (4.6)	5 (4.0)	2 (7.7)	
MTX/FA	37 (24.5)	30 (24.0)	7 (26.9)	
Hysterectomy + CTx	11 (7.3)	9 (7.2)	2 (7.7)	
EMA-CO	8 (5.3)	7 (5.6)	1 (3.8)	
EMA-EP	5 (3.3)	2 (1.6)	3 (11.5) *	
TE/TP	5 (3.3)	1 (0.8)	4 (15.4) *	
Other MTX regimen	6 (4.0)	3 (2.4)	3 (11.5) *	
Unknown	2 (1.3)	2 (1.6)	0 (0.0)	
**Indication for treatment**				0.818 ^b^
Resistance	99 (67.8)	87 (69.0)	12 (60.0)	
Toxicity	11 (7.5)	10 (7.9)	1 (5.0)	
Lack of prior CTx	4 (2.7)	3 (2.4)	1 (5.0)	
No longer urgent	22 (15.1)	18 (14.3)	4 (20.0)	
Other	10 (6.8)	8 (6.3)	2 (10.0)	
**Response to second-line treatment**				0.260 ^b^
No	49 (27.2)	40 (26.1)	9 (33.3)	
Yes	129 (71.7)	112 (73.2)	17 (63.0)	
Unknown	2 (1.1)	1 (0.7)	1 (3.7)	
**hCG at recurrence**	27 (11–206)	26 (10–163)	45 (17–228.5)	0.349 ^a^
**Recurrence**	36 (6.5)	27 (5.7)	9 (11.7)	0.116 ^b^
**Treatment for recurrence**				0.204 ^b^
Hysterectomy	4 (11.1)	3 (11.1)	1 (11.1)	
Hysterectomy + CTx	11 (30.6)	9 (33.3)	2 (22.2)	
Single-agent CTx	7 (19.4)	7 (25.9)	0 (0.0)	
Multiagent CTx	10 (27.8)	5 (18.5)	5 (55.6)	
Other	4 (11.1)	3 (11.1)	1 (11.1)	
**Death**				**<0.001** ^c^
No	535 (97.3)	468 (98.9)	67 (87.0)	
Yes	15 (2.7)	9 (1.9)	6 (13.0)	
**GTN-related death**				**<0.001** ^c^
No	540 (98.2)	472 (99.8)	68 (88.3)	
Yes	7 (1.3)	1 (0.2)	6 (7.8)	
**Age at death (years)**	38.8 ± 9.4	40.4 ± 11.3	38.0 ± 8.9	0.659 ^d^
**Cause of death**				0.646 ^b^
Respiratory failure	4 (26.7)	1 (20.0)	3 (30.0)	
Sepsis	2 (13.3)	1 (20.0)	1 (10.0)	
Treatment resistance	2 (13.3)	0 (0.0)	2 (20.0)	
Other causes	7 (46.7)	3 (60.0)	4 (40.0)	

Footnotes: Data are presented as number (percentage), mean ± standard deviation or median (interquartile range). Bold *p*-values indicate statistical significance. ^a^ Mann–Whitney test; ^b^ Chi-square test; ^c^ Fisher’s exact test; ^d^ Student’s *t*-test; * Statistically significant based on adjusted residuals (*p* < 0.05). **Abbreviations:** CTx = Chemotherapy; EMA-CO = etoposide, methotrexate, dactinomycin, cyclophosphamide, vinblastine; EMA-EP = etoposide, methotrexate, dactinomycin/etoposide, cisplatin; GTN = gestational trophoblastic neoplasia; hCG = Human chorionic gonadotropin; TE/TP = Paclitaxel + Etoposide/Paclitaxel + Cisplatin.

**Table 5:** Multivariable analysis of the association between GTN type and study outcomes.

Outcomes	b (95% CI)	b_adjusted_ (95% CI) *	*p*
**Time to hCG normalization (weeks)**	0.47 (−1.34 to 2.27)	0.26 (−1.92 to 2.44)	0.814
	**RR (95% CI)**	RR_adjusted_ (95% CI) *	***p***
**Response to first-line treatment**	0.92 (0.78–1.08)	0.83 (0.68–1.01)	0.065
**Surgery**	2.01 (1.63–2.48)	1.04 (0.95–1.14)	0.366
**Later pregnancy**	0.36 (0.21–0.61)	0.60 (0.36–1.00)	0.049
	**HR (95% CI)**	HR_adjusted_ (95% CI) *	***p***
**Disease-specific survival**	54.0 (6.85–426)	9.41 (0.70–127)	0.092
**Progression-free survival**	2.10 (1.00–4.46)	1.61 (0.57–4.60)	0.372

**Abbreviation:** b = Regression coefficient; 95% CI = 95% confidence interval; HR = Hazard Ratio; RR = relative risk * Adjusted for parity, number of prior abortions, site of initial treatment, pretreatment hCG level, WHO risk score, type of chemotherapy (single-agent or multiagent), interval between the end of pregnancy and initiation of treatment, definitive histopathological diagnosis, and presence of metastases.

## Data Availability

The data presented in this study are not publicly available due to ethical and privacy restrictions related to patient confidentiality. De-identified data may be made available from the corresponding author upon reasonable request and subject to approval by the Institutional Review Board of Santa Casa de Porto Alegre and applicable ethical regulations.

## Supplementary Materials

The following supporting information can be downloaded at: https://www.mdpi.com/article/10.3390/curroncol33060352/s1, Figure S1: Disease-specific survival according to initial treatment site (GTDC versus outside GTDC); Figure S2: Progression-free survival according to initial treatment site (GTDC versus outside GTDC); Figure S3: Disease-specific survival in the overall cohort; Figure S4: Progression-free survival in the overall cohort; Table S1: FIGO anatomical staging and modified WHO prognostic scoring system for gestational trophoblastic neoplasia (GTN); Table S2: Toxicity of single-agent chemotherapy in GTN; Table S3: Reproductive outcomes according to GTN type; Table S4: Baseline cohort characteristics according to initial treatment site in GTN; Table S5: Clinical characteristics according to GTN type in patients with choriocarcinoma.
