# Supplementary material for: Gestational Trophoblastic Neoplasia Following Hydatidiform Mole and Non-Molar Pregnancy: Clinical and Prognostic Features from a 40-Year Cohort Study at a Reference Center in Southern Brazil
Source: Curr Oncol. 2026 Jun 11;33(6):352. doi: 10.3390/curroncol33060352 (PMC13298583; doi:10.3390/curroncol33060352)
Supplement: Supplementary file 1 [file curroncol-33-00352-s001.zip › Supplementary _Figure_S3_survival_ALL (7).pdf]

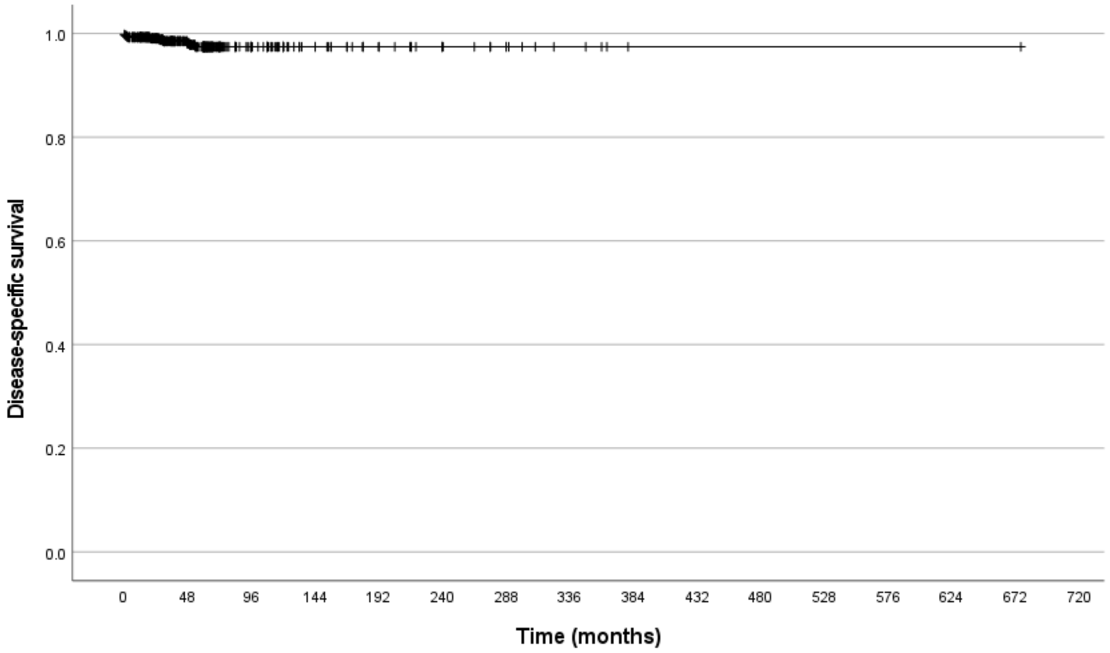

Time (months)	Number at risk	Disease-specific survival
1	546	99.6%
3	540	99.3%
6	528	99.3%
12	505	99.3%
24	409	99.0%
36	331	98.5%
48	288	98.2%
60	244	97.4%
120	52	97.4%
240	18	97.4%
360	3	97.4%

Supplementary Figure S3. Disease-specific survival in the overall cohort
