# Supplementary material for: Gestational Trophoblastic Neoplasia Following Hydatidiform Mole and Non-Molar Pregnancy: Clinical and Prognostic Features from a 40-Year Cohort Study at a Reference Center in Southern Brazil
Source: Curr Oncol. 2026 Jun 11;33(6):352. doi: 10.3390/curroncol33060352 (PMC13298583; doi:10.3390/curroncol33060352)
Supplement: Supplementary file 1 [file curroncol-33-00352-s001.zip › Supplementary_ Table_S1_FIGO(6).pdf]

Supplementary Table S1. **FIGO anatomical staging and risk score system***

**Stage I**	Disease confined to the uterus
**Stage II**	GTN extends outside of the uterus, but is limited to the genital structures (adnexa, vagina, broad ligament)
**Stage III**	GTN extends to the lungs with or without known genital tract involvement
**Stage IV**	All other metastatic sites

*FIGO 2002[9]

Modified WHO Prognostic Scoring System as adapted by FIGO for GTN

**Scores**	**0**	**1**	**2**	**4**
Age (years)	< 40	≥ 40	–	–
Antecedent pregnancy	mole	abortion	term	–
Interval months from index pregnancy	< 4	4 to 6	7 to 12	> 12
Pre-treatment serum hCG (IU/L)	$< 10^3$	10^3–10^4	10^4 to 10^5	$> 10^5$
Largest tumour size (including uterus)	< 3	3 to 4 cm	≥ 5 cm	–
Site of metastases	lung	spleen, kidney	gastrointestinal	liver, brain
Number of metastases	–	1 to 4	5 to 8	> 8
Previous failed chemotherapy	–	–	single drug	≥ 2 drugs
To stage and allot a risk factor score, a patient's diagnosis is allocated to a stage as represented by a Roman numeral I, II, III and IV. This is then separated by a colon from the sum of all the actual risk factor scores expressed in Arabic numerals, i.e. stage II:4, stage IV:9. This stage and score will be allotted for each patient (FIGO 2002) [9] [20]. A score ≤ 6 indicates low risk; > 6 indicates; high risk 7-12; Ultra-high ≥13.				

Abbreviation: GTN=gestacional trophoblastic neoplasia; FIGO= International Federation of Gynecology and Obstetrics; WHO= World Health Organization
