# Supplementary material for: Gestational Trophoblastic Neoplasia Following Hydatidiform Mole and Non-Molar Pregnancy: Clinical and Prognostic Features from a 40-Year Cohort Study at a Reference Center in Southern Brazil
Source: Curr Oncol. 2026 Jun 11;33(6):352. doi: 10.3390/curroncol33060352 (PMC13298583; doi:10.3390/curroncol33060352)
Supplement: Supplementary file 1 [file curroncol-33-00352-s001.zip › Supplementary_Figure_S1_CDT(6).pdf]

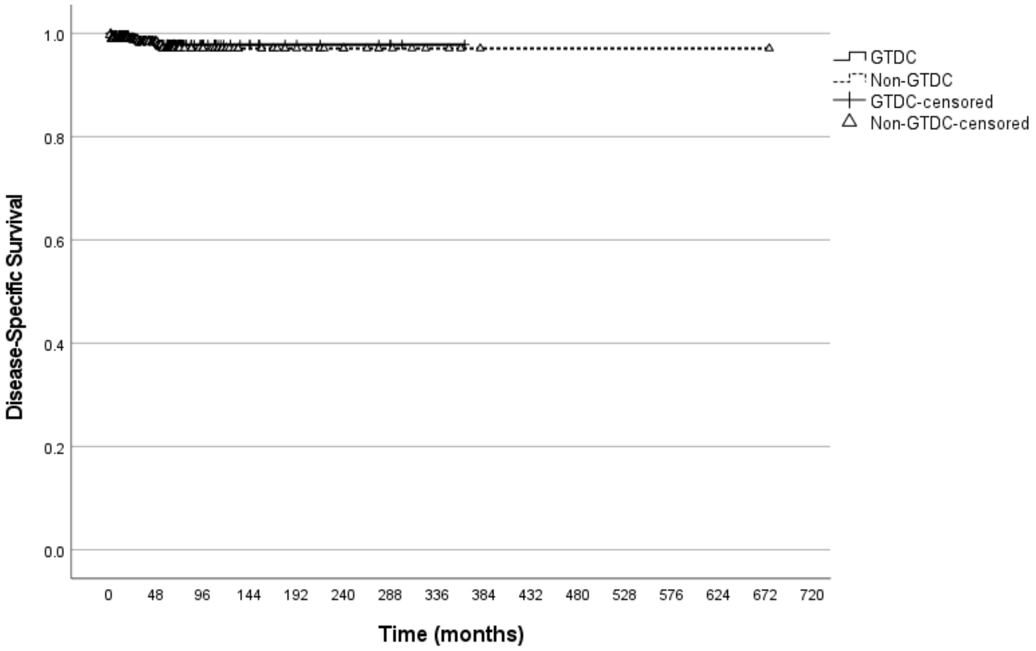


| Time (months) | GTDC | | Outside-GTDC | |
|---|---|---|---|---|
| | Number at risk | Survival probability | Number at risk | Survival probability |
| 1 | 272 | 100% | 274 | 99.3% |
| 3 | 269 | 99.6% | 270 | 98.9% |
| 6 | 261 | 99.6% | 266 | 98.9% |
| 12 | 246 | 99.6% | 258 | 98.9% |
| 24 | 195 | 99.2% | 213 | 98.9% |
| 36 | 149 | 98.6% | 181 | 98.4% |
| 48 | 127 | 97.8% | 160 | 98.4% |
| 60 | 105 | 97.8% | 138 | 97.1% |
| 120 | 14 | 97.8% | 38 | 97.1% |
| 240 | 5 | 97.8% | 13 | 97.1% |
| 360 | 1 | 97.8% | 2 | 97.1% |

**Supplementary Figure S1**. Disease-specific survival according to initial treatment site (log rank test: $\chi^2$=0.21; p=0.647)

**Abbreviation:** GTCD= Gestational Trophoblastic Disease Center
