# Supplementary material for: Gestational Trophoblastic Neoplasia Following Hydatidiform Mole and Non-Molar Pregnancy: Clinical and Prognostic Features from a 40-Year Cohort Study at a Reference Center in Southern Brazil
Source: Curr Oncol. 2026 Jun 11;33(6):352. doi: 10.3390/curroncol33060352 (PMC13298583; doi:10.3390/curroncol33060352)
Supplement: Supplementary file 1 [file curroncol-33-00352-s001.zip › Supplementary_Figure_S2_CDT(6).pdf]

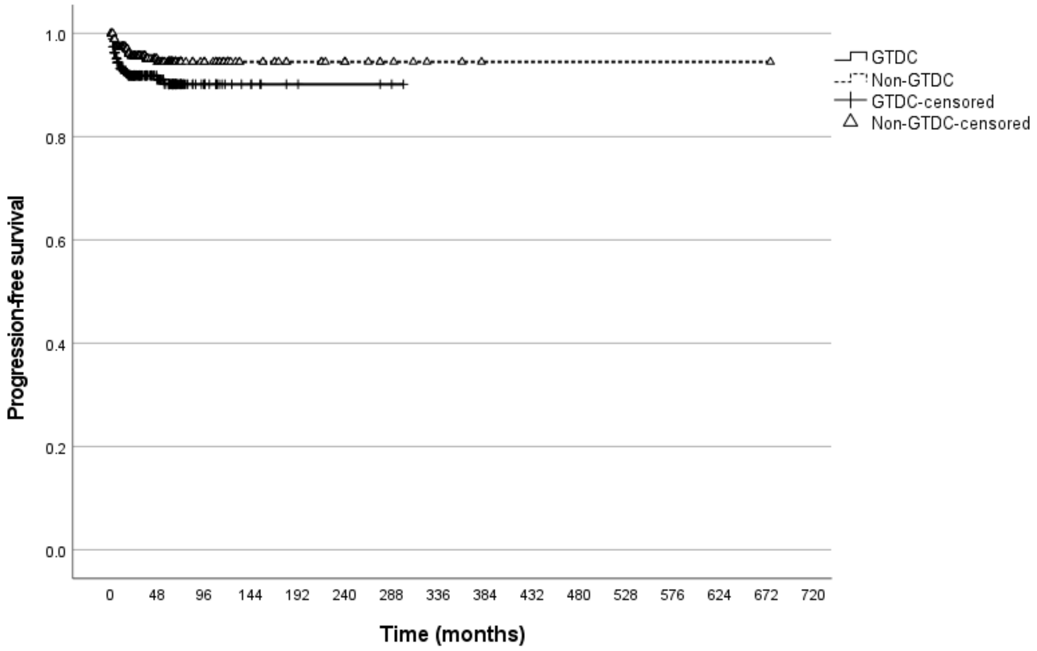

Time (months)	GTDC		Outside GTDC	
	Number at risk	Progression-free Survival probability	Number at risk	Progression-free Survival probability
1	272	99.6%	274	100%
3	266	98.9%	271	98.9%
6	249	95.1%	262	97.4%
12	228	93.2%	252	97.4%
24	175	91.9%	203	95.7%
36	134	91.9%	172	95.1%
48	114	91.1%	152	94.5%
60	95	90.2%	131	94.5%
72	40	90.2%	61	94.5%
84	26	90.2%	50	94.5%
96	22	90.2%	46	94.5%
108	17	90.2%	41	94.5%
120	11	90.2%	33	94.5%
180	4	90.2%	18	94.5%
240	3	90.2%	12	94.5%
300	1	90.2%	5	94.5%
360	0	90.2%	2	94.5%

Supplementary Figure S2. Progression-free survival according to initial treatment site (log rank test: χ^2=3.62; p=0.057).
Abbreviation: GTDC= Gestational Trophoblastic Disease Center
