# Supplementary material for: Gestational Trophoblastic Neoplasia Following Hydatidiform Mole and Non-Molar Pregnancy: Clinical and Prognostic Features from a 40-Year Cohort Study at a Reference Center in Southern Brazil
Source: Curr Oncol. 2026 Jun 11;33(6):352. doi: 10.3390/curroncol33060352 (PMC13298583; doi:10.3390/curroncol33060352)
Supplement: Supplementary file 1 [file curroncol-33-00352-s001.zip › Supplementary_Figure_S4_PFS_ALL(8).pdf]

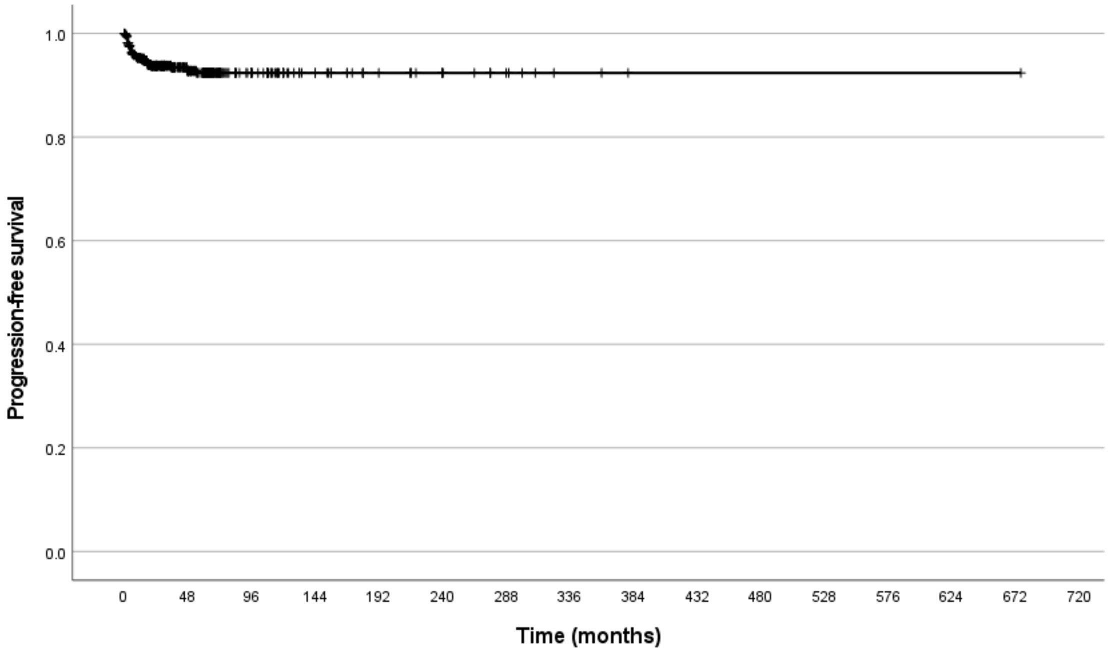

Time (months)	Number at risk	Progression-free survival
1	546	99.8%
3	538	98.2%
6	512	96.8%
12	481	95.3%
24	379	93.8%
36	307	93.5%
48	267	92.8%
60	227	92.4%
72	102	92.4%
84	77	92.4%
96	69	92.4%
108	59	92.4%
120	44	92.4%
180	23	92.4%
240	15	92.4%
300	5	92.4%
360	2	92.4%
420	1	92.4%
480	1	92.4%

Supplementary Figure S4. Disease-specific survival in the overall cohort
