# Supplementary material for: Gestational Trophoblastic Neoplasia Following Hydatidiform Mole and Non-Molar Pregnancy: Clinical and Prognostic Features from a 40-Year Cohort Study at a Reference Center in Southern Brazil
Source: Curr Oncol. 2026 Jun 11;33(6):352. doi: 10.3390/curroncol33060352 (PMC13298583; doi:10.3390/curroncol33060352)
Supplement: Supplementary file 1 [file curroncol-33-00352-s001.zip › Supplementary_Table S5_GTN(8).pdf]

Supplementary Table S5. Clinical characteristics according to GTN type in patients with choriocarcinoma

Variable	Molar GTN (n=23)	Non-molar GTN (n=48)	p-value
**Age (years)**	36.5 ± 11.9	30.6 ± 8.8	**0.022**[a]
**Place of initial treatment**			**0.003**[b]
Reference GTDC	11 (47.8)	6 (12.5)	
Outside GTDC	12 (52.2)	42 (87.5)	
**Follow-up time (months)**	66 (48- 204) [10-348]	51.5 (20-159) [50-364]	0.111[c]
**Pretreatment hCG (IU/L)**	19,106 (3,855–64,105)	53,700 (8,801 – 19,003)	**0.002**[c]
**FIGO stage (2002)**			<0.075[b]
I	15 (65.2)	17 (35.4)	
II	0 (0.0)	5 (10.4)	
III	6 (26.1)	17 (35.4)	
IV	2 (8.7)	9 (18.8)	
**WHO risk score (2002)**			**<0.001**[b]
Low (0–4)	14 (60.9)*	8 (16.7)	
Low (5–6)	3 (13.0)	7 (14.6)	
High (7–12)	5 (21.7)	28 (58.3)*	
Ultra-high ≥ 13	1 (4.3)	5(10.4)	
**Time from last pregnancy to initial treatment (weeks)**	10 (5 - 21)	16 (6-25)	0.445[c]
**Metastasis**			0.052[b]
No	14 (60.9)	16 (33.3)	
Yes	9 (39.1)	32 (66.7)	
**Sites of metastasis**			0.140[b]
Lung	5(21.7)	14 (29.2)	
Vagina	0 (0.9)	2 (4.2)	
Multiple	4 (17.4)	16 (33.3)	
**Surgery**	15 (65.2)	36 (75.0)	0.565[b]
**Type of surgery**			0.131[b]
Hysterectomy	7 (46.7)	19 (52.8)	
Repeat uterine evacuation	3 (20.0)	6 (16.7)	
Hysteroscopy	7 (4.6)	1 (2.0)	
Laparotomy (other indication)	0 (0.0)	7 (19.4)	
Neurosurgery	1 (6.7)	0 (0.0)	
Multiple	4 (26.7)	4 (11.1)	
**Initial CTx regimen**			**0.021**[b]
MTX / FA	12 (52.2)*	6 (12.5)	
Act- D (pulse)	3 (13.0)	11 (22.9)	
EMA-CO	3 (13.0)	19 (39.6)*	
Low-dose EP	1 (4.3)	5 (10.4)	
EMA-EP	0 (0.0)	2 (4.2)	
EMA	0 (0.0)	1 2.1)	
No CTx	1 (4.3)	4 (4.2)	
Other	1 (4.3)	1 (2.1)	
**Response to first-line treatment**			0.294[b]
No	11 (47.8)	14 (29.2)	
Yes	11 (47.8)	32 (66.7)	
No CTx	1 (4.3)	2 (4.2)	
**Time to hCG normalization (weeks)**	17 (8 - 24)	10 (6 – 16)	**0.049**[c]
**Recurrence**			**< 0.001**[b]
No	10 (43.5)	42 (87.5)	
Yes	13 (56.5)	6 (12.5)	
**GTN-related death**			0.261[d]
No	22 (95.7)	41 (85.4)	
Yes	1 (4.34)	6 (12.5)	

Footnotes: Data are presented as number (percentage), mean ± standard deviation or median (interquartile range)

[a] student's t-test; [b] chi-square test; [c] Mann-Whitney test; [d] Fisher's exact test

* Statistically significant based on adjusted residuals (p < 0.05). Bold p-values indicate statistical significance.

Abbreviations: Act-D = actinomycin D; **CTx=** Chemotherapy; EMA= etoposide, methotrexate, actinomycin; EP = etoposide and cisplatin; EMA-CO = etoposide, methotrexate, dactinomycin, cyclophosphamide, vinblastine; EMA-EP = etoposide, methotrexate, dactinomycin/etoposide, cisplatin; GTN = gestational trophoblastic neoplasia; normal; GTDC=gestational trophoblastic disease center; normal hCG=Human chorionic gonadotropin < 5UI/L; MTX/FA= methotrexate/ folinic acid; GTN = gestational trophoblastic neoplasia; GTDC = gestational trophoblastic disease center. WHO= World Health Organization.
