# Supplementary material for: Gestational Trophoblastic Neoplasia Following Hydatidiform Mole and Non-Molar Pregnancy: Clinical and Prognostic Features from a 40-Year Cohort Study at a Reference Center in Southern Brazil
Source: Curr Oncol. 2026 Jun 11;33(6):352. doi: 10.3390/curroncol33060352 (PMC13298583; doi:10.3390/curroncol33060352)
Supplement: Supplementary file 1 [file curroncol-33-00352-s001.zip › Supplementary_Table_S2_GTN(8).pdf]

Supplementary Table S2 – Toxicity of Single-Agent Chemotherapy in GTN

Complication	Methotrexate (n=294)	Actinomycin D (n=171)	p-value
**No toxicity**	149 (50.7)	56 (32.7)	**<0.001**[a]
**Any complication**	145 (49.3)	119 (69.6)	**<0.001**[a]
**Stomatitis**	104 (71.7)	22 (18.5)	**<0.001**[a]
**Nausea/Vomiting or Diarrhea**	32 (22.1)	33 (27.7)	0.358[a]
**Epigastric pain**	11 (7.6)	3 (2.5)	0.121[a]
**Dry eye**	38 (26.2)	4 (3.4)	**<0.001**[a]
**Phlebitis**	0 (0.0)	6 (5.0)	**0.008**[b]
**Pleuritic pain**	32 (22.1)	3 (2.5)	**<0.001**[a]
**Mild hair loss**	5 (3.4)	6 (5.0)	0.551[b]
**Hepatotoxicity**	15 (10.3)	2 (1.7)	**0.009**[a]
**Neutropenia**	8 (5.5)	9 (7.6)	0.673[a]
**Thrombocytopenia**	4 (2.8)	7 (5.9)	0.231[b]
**Severe anemia / blood transfusion**	1 (0.7)	1 (0.8)	1.000[b]
**Fatigue**	10 (6.9)	8 (6.7)	1.000[a]

Footnotes: Values are presented as n (%). All complications in single-agent groups were mild. Bold p-values indicate statistical significance.

[a] Chi-square test; [b] Fisher's exact test.

Abbreviation: GTN = gestational trophoblastic neoplasia.
