# Supplementary material for: Gestational Trophoblastic Neoplasia Following Hydatidiform Mole and Non-Molar Pregnancy: Clinical and Prognostic Features from a 40-Year Cohort Study at a Reference Center in Southern Brazil
Source: Curr Oncol. 2026 Jun 11;33(6):352. doi: 10.3390/curroncol33060352 (PMC13298583; doi:10.3390/curroncol33060352)
Supplement: Supplementary file 1 [file curroncol-33-00352-s001.zip › Supplementary_Table_S3_GTN (7).pdf]

Supplementary Table S3 – Reproductive outcomes according to GTN type

Variable	Total (n=550)	Molar GTN (n=473)	Non-molar GTN (n=77)	p-value[a]
**Subsequent pregnancy**				**<0.001**
No	258 (46.9)	204 (43.1)	54 (70.1)*	
Yes	197 (35.8)	186 (39.3)*	11 (14.3)	
Unknown	95 (17.3)	83 (17.5)	12 (15.6)	
**Type of subsequent pregnancy (n=197)**				**0.002**
Delivery	143 (72.6)	134 (72.0)	9 (81.8)	
Normal evolution	19 (9.6)	19 (10.2)	0 (0.0)	
Miscarriage	23 (11.7)	22 (11.8)	1 (9.1)	
GTD	6 (3.0)	6 (3.2)	0 (0.0)	
Unknown	5 (2.5)	5 (2.7)	0 (0.0)	
Ectopic pregnancy	1 (0.5)	0 (0.0)	1 (9.1)*	
**Reason for no pregnancy (n=258)**				**<0.001**
Hysterectomy	82 (14.9)	56 (11.8.)	26 (33.7)*	
Age/parity	55 (21.3)	49 (24.0)*	6 (11.1)	
Tubal ligation	8 (3.1)	6 (2.9)	2 (3.7)	
IUD	3 (1.2)	3 (1.5)	0 (0.0)	
Unknown	16 (6.2)	15 (7.4)	1 (1.9)	
Synechiae	5 (1.9)	4 (2.0)	1 (1.9)	
No desire	46 (17.8)	44 (21.6)*	2 (3.7)	
Infertility	2 (0.8)	2 (1.0)	0 (0.0)	
Others	1 (0.4)	1 (0.5)	0 (0.0)	
Death	11 (4.3)	4 (2.0)	7 (13.0)*	

Footnotes: Data are presented as number (percentage).

[a] Chi-square test

* Statistically significant based on adjusted residuals ($p < 0.05$). Bold p-values indicate statistical significance.

Abbreviations: GTD = gestational trophoblastic disease; IUD = intrauterine device.
