# Supplementary material for: Gestational Trophoblastic Neoplasia Following Hydatidiform Mole and Non-Molar Pregnancy: Clinical and Prognostic Features from a 40-Year Cohort Study at a Reference Center in Southern Brazil
Source: Curr Oncol. 2026 Jun 11;33(6):352. doi: 10.3390/curroncol33060352 (PMC13298583; doi:10.3390/curroncol33060352)
Supplement: Supplementary file 1 [file curroncol-33-00352-s001.zip › Supplementary_Table_S4_GTN(7).pdf]

Supplementary Table S4 – Baseline Cohort Characteristics by Treatment Site in GTN

Variable	Total (n=550)	GTDC (n=273)	Outside GTDC (n=277)	p-value
**Antecedent pregnancy**				**<0.001[a]**
Complete mole	392 (71.3)	233 (85.3)*	159 (57.4)	
Partial mole	75 (13.6)	24 (8.8)	51 (18.4)*	
Abortion	47 (8.5)	9 (3.3)	38 (13.7)*	
Delivery	27 (4.9)	5 (1.8)	22 (7.9)*	
Ectopic	5 (0.9)	1 (0.4)	4 (1.4)	
Unknown	4 (0.7)	1 (0.4)	3 (1.1)	
**GTN type**				**<0.001[a]**
Molar	473 (86.0)	259 (94.9)	214 (77.3)	
Non-molar	77 (14.0)	14 (5.1)	63 (22.7)	
**FIGO stage**				**<0.001[a]**
I	455 (82.7)	245 (89.7)*	210 (75.8)	
II	12 (2.2)	5 (1.8)	7 (2.5)	
III	66 (12.0)	20 (7.3)	46 (16.6)*	
IV	17 (3.1)	3 (1.1)	14 (5.1)*	
**WHO score**				**<0.001[a]**
≤4	453 (82.4)	251 (91.9)*	202 (72.9)	
5–6	38 (6.9)	12 (4.4)	26 (9.4)*	
7–12	52 (9.5)	9 (3.3)	43 (15.5)*	
≥13	7 (1.3)	1 (0.4)	6 (2.2)	
**Time to initial treatment (weeks)**	8 (5–12) [0–832]	7 (5–10) [0–832]	8 (6–14) [0–572]	**<0.001[b]**
**Follow-up (months)**	49.5 (23–66)	42 (21.5–66)	60 (25–71)	**0.008[b]**
**Final pathology**				**<0.001[a]**
Invasive mole	458 (83.3)	248 (90.8)*	210 (75.8)	
Choriocarcinoma (CCA)	71 (12.9)	17 (6.2)	54 (19.5)*	
PSTT (others)	6 (1.1)	2 (0.7)	4 (1.4)	
ETT (others)	3 (0.5)	2 (0.7)	1 (0.4)	
Same as initial pathology	2 (0.4)	0 (0.0)	2 (0.7)	
Other	10 (1.8)	4 (1.5)	6 (2.2)	
**Initial CTx**				**<0.001[a]**
MTX + FA	294 (53.5)	183 (67.0)*	111 (40.1)	
ACT-D pulse	169 (30.7)	70 (25.6)	99 (35.7)*	
ACT-D 5 days	2 (0.4)	2 (0.7)	0 (0.0)	
EMA-CO	34 (6.2)	4 (1.5)	30 (10.8)*	
MAC III	5 (0.9)	3 (1.1)	2 (0.7)	
Other MTX regimens	9 (1.6)	2 (0.7)	7 (2.5)	
Low-dose EP	6 (1.1)	2 (0.7)	4 (1.4)	
EMA-EP	3 (0.5)	0 (0.0)	3 (1.1)	
EMA	3 (0.5)	0 (0.0)	3 (1.1)	
No treatment	23 (4.2)	7 (2.6)	16 (5.8)	
Other	2 (0.4)	0 (0.0)	2 (0.7)	
**Response to first treatment**	402 (73.1)	188 (68.9)	214 (77.3)	**0.045[a]**
**Time to hCG normalization (weeks)**	9 (6–14)	8 (5–40)	9 (6–13)	0.910[b]
**Metastases**	99 (18.0)	31 (11.4)	68 (24.5)	**<0.001[a]**
**Surgery**	203 (36.9)	60 (22.0)	143 (51.6)	**<0.001[a]**
**Type of surgery**				**0.014[a]**
Hysterectomy	82 (40.4)	33 (55.0)*	49 (34.3)	
Repeat curettage	68 (33.5)	10 (16.7)	58 (40.6)*	
Hysteroscopy	8 (3.9)	4 (6.7)	4 (2.8)	
Laparotomy (other causes)	17 (8.4)	6 (10.0)	11 (7.7)	
Uterine artery embolization	4 (2.0)	1 (1.7)	3 (2.1)	
Pulmonary resection	1 (0.5)	1 (1.7)	0 (0.0)	
Neurosurgery	2 (1.0)	1 (1.7)	1 (0.7)	
Multiple	21 (10.3)	4 (6.7)	17 (11.9)	
**Recurrence**	36 (6.5)	23 (8.4)	13 (4.7)	0.172[a]
**hCG at recurrence**	27 (11–206)	17 (10–93)	87 (17–240.5)	0.140[b]
**Treatment of recurrence (n=36)**				0.420[a]
Hysterectomy	4 (11.1)	4 (17.4)	0 (0.0)	
Hysterectomy + CTx	11 (30.6)	6 (26.1)	5 (38.5)	
Single-agent CTx	7 (19.4)	5 (21.7)	2 (15.4)	
Multiagent CTx	10 (27.8)	5 (21.7)	5 (38.5)	
Other	4 (11.1)	3 (13.0)	1 (1.7)	

Footnotes: Data are presented as number (percentage) or median (interquartile range). Bold p-values indicate statistical significance; [a] Chi-square test; [b] Mann-Whitney test

Abbreviation: CCA = choriocarcinoma; CTx= Chemotherapy; EP = etoposide and cisplatin; EMA= Etoposide, methotrexate, actinomycin; EMA-CO = etoposide, methotrexate, dactinomycin, cyclophosphamide, vinblastine; EMA-EP = etoposide, methotrexate, dactinomycin/etoposide, cisplatin; ETT = epithelioid trophoblastic tumor; FIGO=International Federation of Gynecology and Obstetrics; GTN = gestational trophoblastic neoplasia; normal hCG=Human chorionic gonadotropin < 5UI/L; IQR= interquartile range; MAC III = MTX + Act-D + cyclophosphamide; MTX/FA= methotrexate, folinic acid; Act-D = actinomycin D; PSTT = placental site trophoblastic tumor; WHO= World Health Organization.

;
